# Decoding the Tumor Microenvironment of Myoepithelial Cells in Triple-Negative Breast Cancer Through Single-Cell and Transcriptomic Sequencing and Establishing a Prognostic Model Based on Key Myoepithelial Cell Genes

**DOI:** 10.1155/ijog/6454413

**Published:** 2025-05-06

**Authors:** Xiaocheng Yu, Ye Tian, Rui Zhang, Yong Yang

**Affiliations:** Department of Thyroid and Breast Surgery, Wuhan No.1 Hospital, Tongji Medical College, Huazhong University of Science and Technology, Wuhan, China

**Keywords:** myoepithelial cells, prognosis, TNBC

## Abstract

**Background:** Triple-negative breast cancer (TNBC) is an aggressive subtype with high malignancy, rapid progression, and a poor 5-year survival rate of ~77%. Due to the lack of targeted therapies, treatment options are limited, highlighting the urgent need for novel therapeutic strategies. Myoepithelial cells (MECs) in the tumor microenvironment may significantly influence TNBC development and progression.

**Methods:** This study used single-cell RNA sequencing to compare the MEC gene expression in the normal versus TNBC tissues. TNBC-associated MECs showed increased expression of proliferation- and immune-related genes (e.g., FDCSP, KRT14, and KRT17) and decreased expression of inflammatory and extracellular matrix-related genes (e.g., CXCL8, SRGN, and DCN). Copy number variation and pseudotime analyses revealed genomic alterations and phenotypic dynamics in MECs. A CoxBoost-based prognostic model was developed and validated across 20 survival cohorts, integrating immune profiling, pathway enrichment, and drug sensitivity analyses. Mendelian randomization identified TPD52 as a TNBC risk–associated gene. siRNA knockdown of TPD52 was performed in TNBC cell lines to evaluate its effects on proliferation and migration.

**Results:** TNBC MECs displayed significant changes in the gene expression and genomic integrity, impacting immune responses and tumor invasion. The prognostic model effectively predicted 1-, 3-, and 5-year survival outcomes, stratifying high-risk patients with enriched cell cycle and DNA replication pathways, reduced immune checkpoint expression, and chemotherapy resistance. TPD52 was identified as a tumor-promoting gene, and its knockdown suppressed TNBC cell proliferation and migration.

**Conclusion:** This study highlights MECs' role in TNBC progression, provides a CoxBoost prognostic model for personalized treatment, and identifies TPD52 as a potential therapeutic target for TNBC intervention.

## 1. Introduction

Based on the 2022 global cancer statistics, breast cancer (BRCA) ranks as the second most common cancer globally, with around 2.3 million new cases, making up 11.6% of all cancer diagnoses [1]. It ranks as the fourth most common cause of deaths related to cancer, responsible for about 666,000 fatalities (6.9% of all cancer deaths). BRCA is the most diagnosed cancer and the top cause of cancer-related deaths among women worldwide, ranking first in incidence in 157 countries and first in mortality in 112 countries. Triple-negative breast cancer (TNBC) comprises 15–20% of all BRCA cases and is characterized by the absence of the estrogen receptor (ER) and progesterone receptor (PR) expressions, as well as a lack of human epidermal growth factor receptor 2 (HER2) overexpression or amplification [2]. TNBC is widely acknowledged as the most aggressive subtype, related with the poorest clinical outcomes [3].

Advances in single-cell technologies have supplied novel understandings into the tumor heterogeneity and microenvironment of TNBC [4]. Through rigorous single-cell analyses, we can gain a more comprehensive insight of the signaling cascades, metabolic states, and intercellular interactions among distinct cellular subpopulations [5]. These studies are expected to elucidate the underlying regulatory mechanisms involved in TNBC development, thereby facilitating breakthroughs in personalized therapeutic strategies [6]. In summary, investigating the interplay between myoepithelial cells (MECs) and TNBC is pivotal for unraveling the mechanisms of tumor initiation and progression and for advancing individualized treatment approaches.

MECs play a dual role in the initiation and progression of BRCA. Under normal conditions, MECs act as a critical barrier by structurally isolating luminal epithelial cells (LECs) from the surrounding stroma [7–9]. Additionally, MECs secrete antiangiogenic factors and protease inhibitors, maintaining LEC polarity and tissue architectural integrity, thereby effectively suppressing tumor initiation and progression [10–12]. However, within certain cancerous microenvironments, MECs can undergo functional abnormalities and secrete protumorigenic chemokines, such as CXCL12 and CXCL14, which interact with receptors on cancer cells to enhance their proliferation and invasive behaviors [13–16]. As the tumor progresses, the number of MECs decreases, and their protective functions are significantly compromised, contributing to increased tumor cell invasiveness and poorer patient prognosis [17]. This phenomenon is particularly evident in TNBC, where the loss of MEC function may accelerate the transition from noninvasive lesions to invasive phenotypes. Therefore, understanding the role and mechanisms of MECs in BRCA, especially in TNBC, is crucial for elucidating tumor biology and developing novel therapeutic strategies and predictive models.

## 2. Materials and Methods

### 2.1. Data Acquisition and Processing of TNBC Samples From Public Databases

We systematically acquired and preprocessed data for TNBC samples and corresponding controls from public databases to elucidate the molecular characteristics of this disease. Firstly, using the GEOquery R package [18–20], we downloaded two single-cell RNA-seq datasets from the GEO database—GSE161529 (69 samples) and GSE268662 (9 samples)—which together provided expression profiles for 15 TNBC samples. In parallel, gene expression data (expressed as transcripts per million, TPM) and somatic mutation data processed by MuTect2 were obtained for BRCA patients from the TCGA-BRCA cohort via the UCSC Xena browser (https://xenabrowser.net/datapages/) [21, 22]. To construct a comprehensive survival analysis dataset, we further downloaded nine additional microarray datasets from GEO (GSE9893, GSE11121, GSE17705, GSE20685, GSE22219, GSE25055, GSE42568, GSE48390, and GSE162228) using the GEOquery R package. After rigorous quality control, only samples with complete survival information were retained, resulting in a final survival dataset comprising 1068 cases from TCGA along with the samples from the aforementioned GEO datasets.

During data processing, we first employed the ComBat algorithm from the SVA R package to merge two GEO microarray datasets and nine GEO clinical information datasets, thereby constructing two integrated datasets—meta-GEO and clinical-GEO [23]. This strategy was implemented to minimize batch effects resulting from nonbiological technical biases across different datasets. For the scRNA-seq data, preprocessing was performed using the Seurat package (version 4.3.0) [23]. Specifically, each sample was imported using the Read10X function, and Seurat objects were created with the parameters min.cells = 3 and min.features = 200. Subsequently, strict quality control measures were applied, retaining cells with 200–5000 detected genes and ensuring that mitochondrial gene expression remained within 0%–15%. These procedures provided high-quality, low-noise baseline data for subsequent analyses [24].

### 2.2. Integrated Single-Cell Analysis: Dimensionality Reduction, Clustering, Cell Annotation, and Differential Analysis

Single-cell RNA sequencing data were initially normalized with the “LogNormalize” method via the NormalizeData function. Subsequently, the top 2000 highly variable genes were identified using the FindVariableFeatures function. Principal component analysis (PCA) was then carried out on these genes to reduce the dimensionality of the dataset. To address potential batch effects across samples, we integrated the data using the Harmony package in R. Cells were clustered at a resolution of 0.8 using the FindClusters and FindResolution functions to group them by gene expression similarity, identifying distinct populations. The results were visualized with UMAP, projecting the data onto a two-dimensional plane. For cell annotation, we assigned cell types based on previously reported marker genes and literature evidence. Luminal cells were identified based on the expression of KRT19, KRT18, and KRT8; MECs were annotated using KRT17, KRT14, and KRT5; and myeloid cells were characterized by CD68, CD163, and CSF1R. T cells were distinguished by CD3D, CD3E, and CD8A, while natural killer cells were recognized using GNLY and NKG7. In addition, B cells were labeled with CD79A, MS4A1, and CD19; endothelial cells were identified with PECAM1 and VWF; and fibroblasts were annotated using PDGFRA, DCN, and PDGFRB. Additionally, the differential gene expression between the tumor and normal samples across the seven cell types was assessed using the Wilcoxon test via the Limma package (*p* < 0.05) [25].

### 2.3. Copy Number Variation (CNV) Assessment in MECs

We utilized the InferCNV package (version 1.14.2) to calculate CNVs in all MECs, using T/NK cells as the reference [26]. Through *K*-means clustering, we identified a subgroup of MECs demonstrating significant chromosomal copy number alterations and classified them as tumor cells. Subsequently, CNV scores were calculated according to the established methodologies from previous studies [27, 28].

### 2.4. Pseudotime Analysis of Malignant Tumor Cells in TNBC

Trajectory analysis was carried out using Monocle (version 2.26.0) to elucidate the cellular changes occurring during the differentiation of various TNBC cells [29]. Initially, a monocle object was established with the “newCellDataSet” function. Subsequently, highly variable genes were filtered and the “DDRTree” algorithm was applied for dimensionality reduction to generate pseudotemporal trajectories. The “differentialGeneTest” function was used to identify genes with differential expressions along pseudotime, which were then visualized in a pseudotime heat map, after which they were ordered based on their pseudotemporal progression [30].

### 2.5. High-Dimensional Weighted Gene Coexpression Network Analysis (hdWGCNA) Analysis

To create a scale-free network at the single-cell level, we employed hdWGCNA with the R package hdWGCNA (version 0.1.1.9010) [31]. First, a threshold was determined for fitting the scale-free topology model; a value greater than 0.8 was selected to ensure the network retained its scale-free characteristics. Next, a soft-threshold power of 5 was chosen to optimize network connectivity, a parameter that affects the strength of relationships between genes and determines the formation of coexpression modules or clusters. Finally, Gene Ontology (GO) biological process enrichment analysis was conducted on the identified modules.

### 2.6. Construction and Evaluation of the Prognostic Model

We employed multiple modeling algorithms, including Lasso regression, elastic net, ridge regression, stepwise Cox regression, and CoxBoost [32, 33]. Lasso regression was implemented with the glmnet package by specifying the family parameter as “cox” and setting the alpha parameter to 1. Then, 10-fold crossvalidation was performed with the cv.glmnet function to select the optimal *λ* value. From the training results, the model coefficients corresponding to the optimal *λ* and their associated feature names were extracted, retaining only those genes with nonzero coefficients. Similarly, both elastic net and ridge regression were executed using the glmnet package. For elastic net, the alpha parameter was varied between 0 and 1 (using values such as 0.1–0.9), while for ridge regression, the alpha parameter was fixed at 0. For stepwise Cox regression, a multivariate Cox model was first constructed using the coxph function, and then, stepwise regression analysis was performed with the stepAIC function, employing directional options that included “both,” “forward,” and “backward.” Regarding the CoxBoost model, we initially optimized the penalty parameter using the optimCoxBoostPenalty function. Subsequently, crossvalidation was conducted with the cv.CoxBoost function to determine the optimal number of boosting steps. Finally, the CoxBoost function was applied, incorporating both the optimal number of steps and the penalty parameter, to build the final model. The coefficients and their corresponding genes were then extracted using the coef function or obtained from the regression coefficient slot of the respective models.

### 2.7. Risk Score (RS) Calculation and Model Evaluation

We computed the linear combination of gene expression data and model coefficients to create a RS for each sample. To assess the performance of the prognostic models, area under the curve (AUC) and receiver operating characteristic (ROC) curves were employed as evaluation metrics. The timeROC package was utilized to calculate the 1-year, 3-year, and 5-year AUC values for multiple models [34]. In addition, univariate Cox analysis was performed via the coxph function to assess the hazard ratio (HR) of the RS computed by the top-ranked algorithm across different datasets. To further bolster the robustness of our findings, meta-analysis using the inverse variance method was conducted on the univariate Cox survival analysis results, with the natural logarithm of the HR serving as the primary measure to evaluate the prognostic value of the model. Finally, Kaplan–Meier (KM) survival analysis was carried out using the survival package. The optimal cutoff for stratifying the samples into the high-risk and low-risk groups was determined using the survminer package [35], ensuring that the minimum proportion for either group was no less than 0.3. The survfit function was then employed to perform the log-rank test, assessing the significance of the survival differences between the two risk groups.

### 2.8. Different Tumor Immune Microenvironment Patterns With RS

We employed TIMER, CIBERSORT-ABS, QUANTISEQ, MCPCOUNTER, CIBERSORT, XCELL, and EPIC algorithms to calculate the proportions of tumor-infiltrating immune cells [36–39]. The differences in these proportions between the high- and low-risk groups were subsequently compared. Additionally, we utilized the R packages ggplot2, ggpubr, and limma to statistically analyze the expression levels of 79 commonly reported immunosuppressive molecules associated with immune checkpoint inhibitors (ICIs). Differences in immune, stromal, and tumor purity scores between the two risk groups were evaluated using the ESTIMATE algorithm. To further delineate the functional and biological process differences between these groups, we conducted Gene Set Enrichment Analysis (GSEA) on Kyoto Encyclopedia of Genes and Genomes (KEGG) gene sets. The top five notable pathways from each analysis are manifested [38, 40].

### 2.9. Drug Sensitivity Analysis Between High- and Low-Risk Groups

The development of novel therapeutics continues to be a critical focus in BRCA treatment. In this study, we employed the R package pRRophetic to estimate the half-maximal inhibitory concentration (IC50) of ordinarily used drugs in BRCA patients [39, 41]. Based on patient risk stratification (*p* < 0.001), we identified potential drugs that exhibited striking differences in sensitivity between the high- and low-risk groups. Boxplots were subsequently generated to visually represent these differences.

### 2.10. Mendelian Randomization (MR) Analysis Was Employed to Identify Key Genes

In this research, we applied MR to investigate the causal relationship between target gene expression levels and BRCA prognosis [42]. The inverse-variance weighted (IVW) method was used to estimate the causal effect of gene expression on prognosis. All analyses were conducted using R (v4.2.0) with the TwoSampleMR, MendelianRandomization, and MRPRESSO packages [43].

### 2.11. Immunohistochemistry

TNBC (tumor) samples and the corresponding normal paired nontumor tissue (ZL-Brc3N961) were procured from ShangHai Zhuoli Biotech Company (China). ShangHai Zhuoli Biotech Company Ethics Committee approved the experiments (Ethics Approval Number: SHLLS-BA-22101102). Subsequently, immunohistochemical (IHC) staining was employed to assess the protein expression of TPD52, utilizing the anti-TPD52 antibody provided by BIOSS (China).

### 2.12. Cell Culture

The MDA-MB-231 and SUM159PT (cancer cell lines of human breast) were obtained from ATCC and cultured in HyClone's DMEM with 10% Lonsera FBS and 1% antibiotic–antimycotic solution. They were cultivated at 37°C in a humidified 5% CO_2_ atmosphere.

### 2.13. Western Blot

Total protein extraction from cells was implemented using RIPA buffer (Beyotime, China), supplemented with phosphatase and protease inhibitors. Western blot analysis was conducted according to a standardized protocol. The primary antibodies used in this research were anti-TPD52 (BIOSS, China) and anti-GAPDH (Proteintech, China).

### 2.14. Cell Transfection

Transfection of small interfering RNA (siRNA) was performed using Lipofectamine 3000 (Invitrogen, Shanghai, China) according to the manufacturer's protocol. Following a 48-h incubation, the cells were washed and subsequently employed in further experimental procedures. The specific siRNA sequences targeting TPD52 were as follows: 5⁣′-ACUUGGAAUCAAUUCUCUA-3⁣′, 3⁣′-AAACUUGGAAUCAAUUCUCUACA-5⁣′.

### 2.15. CCK8 Methods

MDA-MB-231 and SUM159PT cells were seeded at 1 × 10^5^ cells per well in 6-well plates and transfected with either NC-siRNA or TPD52-targeting siRNAs. After 48 h, 1500 cells were moved to 96-well plates and cultured with the respective siRNAs for 0, 24, 48, or 72 h. Cell viability was assessed by adding CCK8 solution for 2 h and measuring the optical density at 450 nm.

### 2.16. Colony Formation Experiment

To assess the effect of TPD52 expression on human BRCA cell proliferation, transfected MDA-MB-231 and SUM159PT cells (2000 per well) were seeded into 6-well plates, and colonies were counted after 12 days.

### 2.17. Transwell Assay

The migration assay used Transwell chambers with MDA-MB-231 and SUM159PT cells seeded at 3 × 10^4^ cells into 200-*μ*L serum-free DMEM in the upper chamber. The lower chambers contained DMEM with 10% fetal bovine serum. After 36 h, the inner chambers were cleaned, and cells on the membrane underside were fixed with 4% formaldehyde, stained with crystal violet, and examined microscopically.

## 3. Results

### 3.1. Characterization of Cellular Heterogeneity and Gene Expression Alterations in TNBC

**Figure 1:**
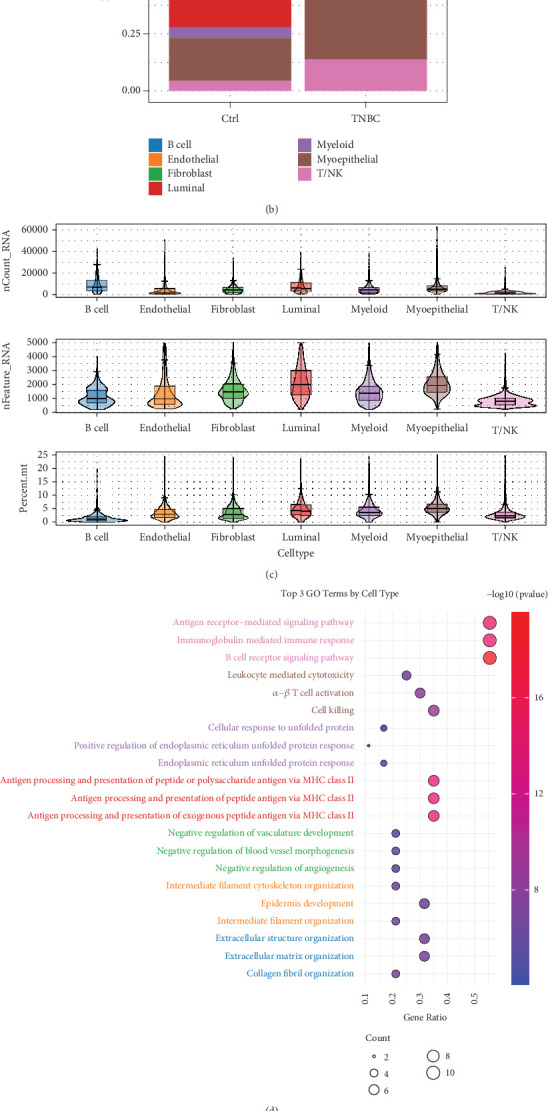
Cellular landscape and molecular features of the seven identified cell subtypes in triple-negative breast cancer (TNBC). (a) Box plots showing the gene expression metrics (nCount, gene count, and mitochondrial gene ratio) for the seven identified cell subtypes in TNBC tissue. (b) The top three Gene Ontology (GO) enrichment pathways identified in each of the seven distinct cell subtypes. (c) UMAP visualization of the seven cell subtypes in TNBC and normal control (NC). (d) bar plots showing the differences in the proportions of the seven cell subtypes between TNBC and NC samples. (e) Differential gene expression analysis for the seven cell subtypes between normal and TNBC tissues, highlighting the top five significantly differentially expressed genes for each subtype.

To better elucidate the cellular heterogeneity of TNBC, we analyzed single-cell sequencing data from 15 TNBC patients and 10 normal breast tissue samples. After rigorous quality control and filtering—and using well-established marker annotations—we characterized seven distinct cell types (Figure 1a: B cells, endothelial cells, fibroblasts, luminal cells, myeloid cells, MECs, and T/NK cells, each defined by specific markers). We observed significant differences in cell clustering between the TNBC and control groups. Notably, fibroblasts, luminal cells, and subepithelial cells exhibited unique distribution and aggregation patterns under TNBC conditions. A stacked bar chart (Figure 1b) depicts the relevant proportions of different cell types between the control and TNBC groups demonstrating shifts in the fractions of specific populations, such as fibroblasts, acinar cells, and subepithelial cells. These changes highlight the potential significance and functional alterations of these cell types within the TNBC microenvironment.

Figure 1c illustrates the gene expression levels and mitochondrial gene ratios in various cell types—including B cells, endothelial cells, and fibroblasts—in TNBC samples. Moreover, Figure 1d represents a bubble plot summarizing the results of GO enrichment analyses for each cell type. These results demonstrate that the gene expression profiles are enriched in diverse biological processes. For instance, myeloid cells display significant enrichment in GO terms associated with cell killing, T cell activation, and cytotoxicity—findings that align with the involvement of myeloid subpopulations (including monocytes, macrophages, neutrophils, eosinophils, basophils, and dendritic cells) in mediating cytolytic activities. In contrast, fibroblasts are enriched in GO terms related to extracellular matrix organization and collagen formation, highlighting the distinct biological functions of different cell types in the TNBC microenvironment.

Finally, Figure 1e portrays the log2 fold changes of differentially expressed genes (DEGs) between the TNBC and control groups across different cell types. Substantial gene expression alterations were observed in fibroblasts, luminal cells, and B cells. For example, in fibroblasts, genes associated with the extracellular matrix (such as MMP3 and COL1A1) were upregulated, whereas in B cells, genes linked to immune responses (such as IGLC2 and IGHG3) were elevated. These findings suggest that both immune responses and extracellular matrix remodeling are intricately linked to the incidence and progression of TNBC.

### 3.2. Integrative CNV and Pseudotime Analyses Reveal Genomic Instability and Temporal Evolution of Tumor Cell States in TNBC

**Figure 2:**
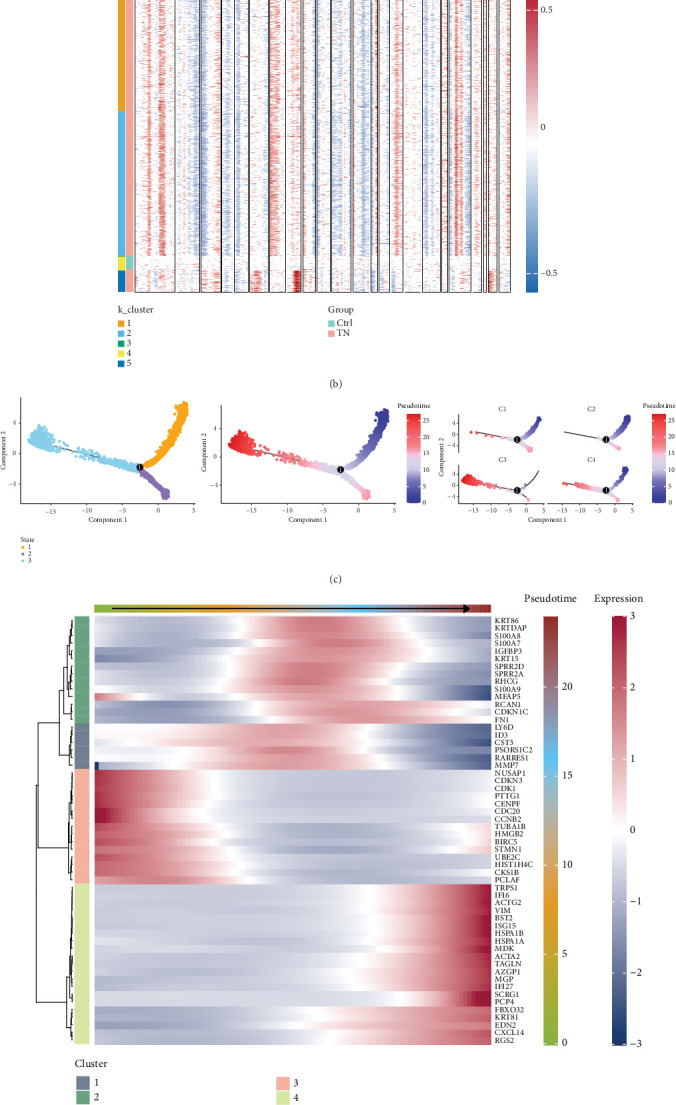
Genomic alterations in myoepithelial cells from TNBC. (a) Heatmap depicting the landscape of copy number variations (CNVs) in myoepithelial cells, using T/NK cells as a reference. (b) *K*-means clustering based on CNVs, highlighting the similarities between TNBC myoepithelial cells in Cluster 4 and T/NK cells. (c) Monocle-based trajectory predictions of CRC tumor cell subpopulations. (d) Classification of pseudotime-dependent genes into four main categories and the associated pathway enrichment. (e) Heatmap showing the marker gene expression across different branches, annotated into four major clusters.

Figure 2a displays the genomic CNV results obtained via inferCNV analysis, revealing potential genomic instability of MECs in the TNBC group, suggesting that MECs also have malignant potential.  Figure 2b shows a gene expression heat map (organized by samples and genes), which highlights pronounced differences in CNV profiles between MECs in the tumor and normal samples. Notably, Cluster 4 exhibits significant overlaps with normal samples, suggesting a considerable level of normal cell contamination within the tumor samples. Therefore, we selected Clusters 1, 2, 3, and 5 for further analysis.

Subsequently, pseudotime analysis was used to infer the developmental sequence of tumor cell states across the four identified clusters. The results indicate that subclusters C1 and C2 are predominantly situated at the early stage of the pseudotime trajectory, whereas subcluster C3 mainly appears during the later stage (Figure 2c). This temporal pattern implies a potential progression in myoepithelial states, with C1 and C2 manifesting less malignant phases and C3 manifesting a more progressive or aggressive state.

Figure 2d presents a heat map illustrating the changes in key gene expression among various clusters across groups, while Figure 2e depicts the expression dynamics of the six most significantly different genes along the pseudotime trajectory—from initiation to termination. In particular, the genes VIM and ACTA2 show elevated expression in cells positioned at later pseudotime stages, suggesting that they may play critical roles in the later phases of cell state transition.

### 3.3. Identification of Core Genes in Tumor Clusters in MECs Via hdWGCNA

**Figure 3:**
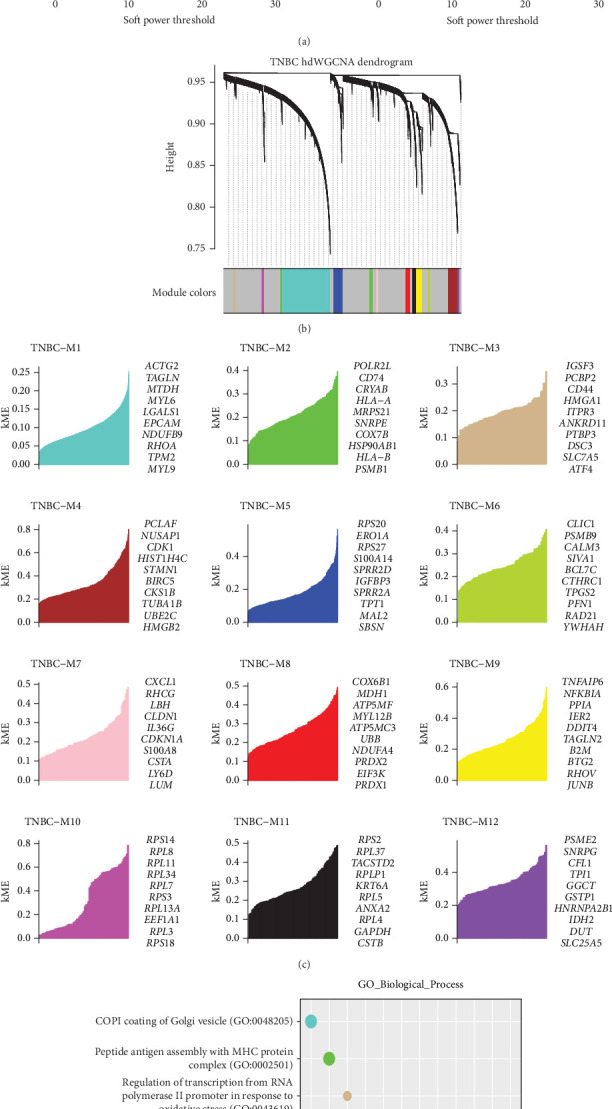
Identification of coexpression modules and prognostic hub genes in TNBC. (a) Weighted gene coexpression network analysis (WGCNA) of TNBC cells. (b) Visualization of the coexpression network structure across different modules. (c) Ranking of the top 10 eigengenes for each module based on module eigengene connectivity (kME). (d) GO enrichment analysis of highly enriched genes within the 16 TNBC coexpression modules.

hdWGCNA was employed to identify core molecular features in tumor clusters by constructing scale-free networks (soft threshold = 5), yielding 12 optimally connected gene modules (Figures 3a, 3b, and 3c). GO enrichment analysis (Figure 3d) then allowed us to delineate the key biological processes associated with these 12 TNBC gene modules. Specifically, the TNBC-M1 module was predominantly enriched in COPI coating of Golgi vesicles; the TNBC-M5, -M10, and -M11 modules were mainly enriched in SRP-dependent cotranslational protein targeting to membranes; the TNBC-M2 module was chiefly enriched in peptide antigen assembly with the MHC protein complex; and the TNBC-M3 module was enriched in controlling transcription from the RNA polymerase II promoter in response to oxidative stress. Further, we select the module containing the most genes, and Module 1 is further analyzed.

### 3.4. Prognostic Model Construction and Validation Using the CoxBoost Algorithm

**Figure 4:**
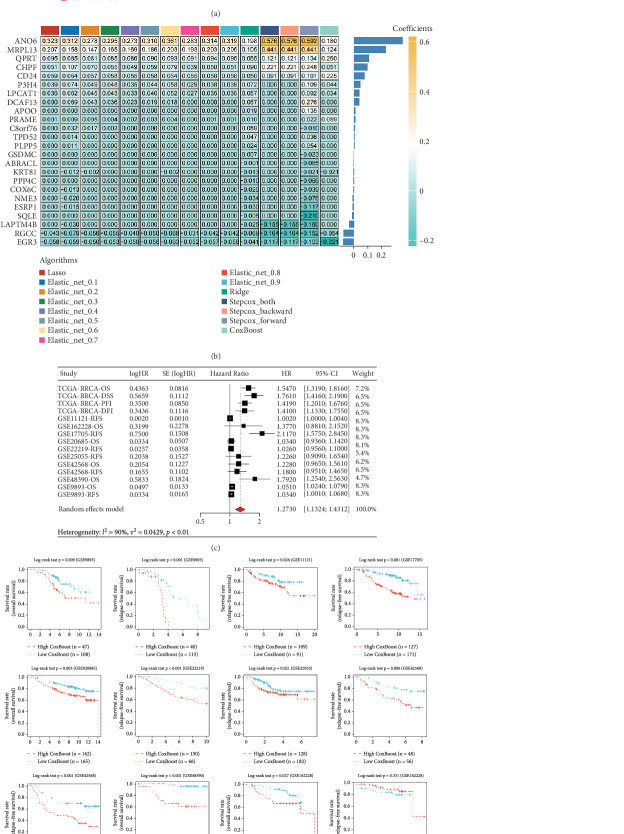
Performance comparison of prognostic regression models. (a) Heatmap demonstrating the mean AUCs (1-year, 3-year, and 5-year) for Lasso regression, elastic net, ridge regression, stepwise Cox regression, and CoxBoost models across 15 cohorts. (b) Heatmap illustrating the regression coefficients of the input genes across various prognostic models. (c) Univariate Cox regression analysis of the risk scores combined with meta-analysis. (d) Kaplan–Meier survival analysis across 20 survival cohorts from 12 datasets.

We employed multiple algorithms to construct prognostic models and comprehensively evaluated them based on the average AUC at 1, 3, and 5 years. Ultimately, the CoxBoost model was determined to be the optimal algorithm (Figure 4a). Detailed comparative analyses revealed that the CoxBoost model consistently achieved exceptional average AUC values at all three time points, clearly distinguishing itself from the others.

A heat map (Figure 4b) illustrates the regression coefficients of the input genes across different prognostic models, providing an intuitive visualization of each gene's contribution. This approach has been instrumental in guiding subsequent model optimization and gene selection. In Figure 4c, we present the univariate Cox regression analysis and its meta-analysis results for the RSs calculated using the optimal CoxBoost model. The analysis confirms that our prognostic model is consistently identified as a risk factor across multiple datasets and various survival endpoints, demonstrating its high predictive accuracy and generalizability.

Finally, as shown by KM analysis in Figure 4d, among 16 distinct cohorts derived from 11 datasets—including overall survival (OS), disease-specific survival (DSS), recurrence-free survival (RFS), progression-free interval (PFI), and disease-free interval (DFI) cohorts—the high-risk group manifested observably poorer prognoses compared to the low-risk group. This finding further validates the clinical reliability and practical applicability of the CoxBoost model for prognostic assessment, thereby providing a robust foundation for future study and clinical applications.

### 3.5. Immune Landscape and Functional Enrichment Analyses in High- and Low-Risk Groups

**Figure 5:**
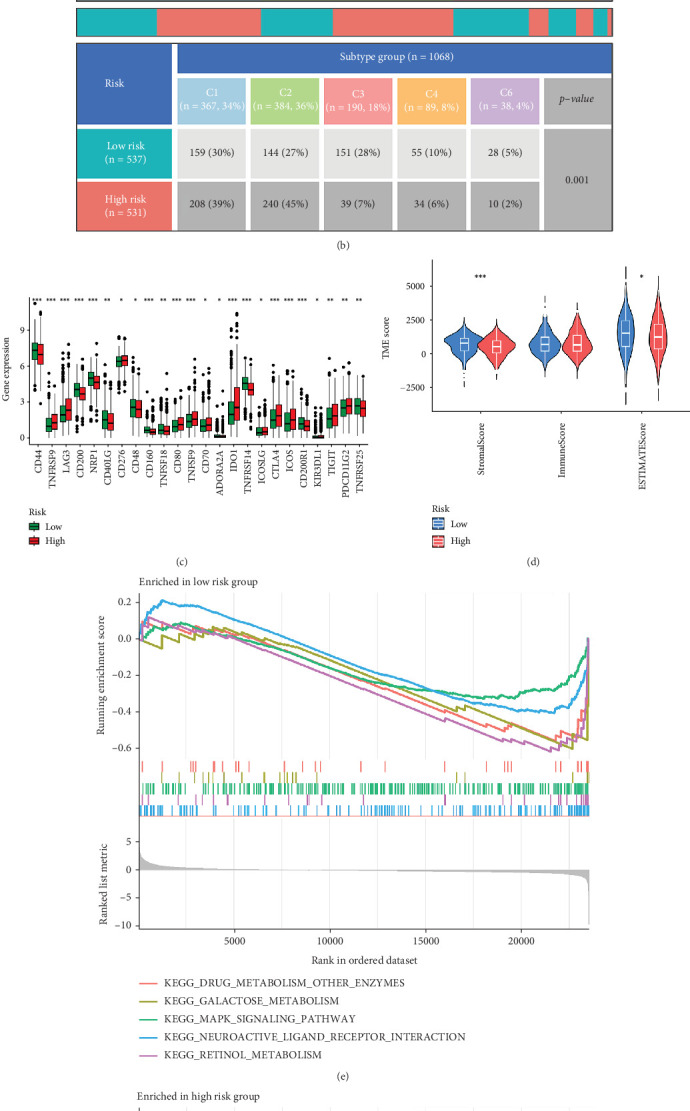
Immune landscape analysis in high- and low-risk TNBC groups. (a) Heatmap comparing immune cell infiltration between the high- and low-risk groups using TIMER, CIBERSORT, CIBERSORT-ABS, QUANTISEQ, MCPCOUNTER, xCell, and EPIC algorithms. (b) Risk and subtype group comparisons in the TCGA dataset. (c) Differential expression analysis for immune checkpoint genes between the high- and low-risk groups (⁣^∗^*p* < 0.05, ⁣^∗∗^*p* < 0.01, and ⁣^∗∗∗^*p* < 0.001). (d) Comparison of immune, stromal, and tumor purity scores between the two risk groups. (e) GSEA enrichment analysis results for the low-risk group. (f) GSEA enrichment analysis results for the high-risk group.

Figure 5a illustrates significant differences in immune cell expression between the high- and low-risk groups as determined by several algorithms, including TIMER, CIBERSORT, CIBERSORT-ABS, QUANTISEQ, MCPCOUNTER, XCELL, and EPIC. Based on the RS, 1068 TCGA samples were stratified into two groups, comprising 531 samples in the high-risk group and 537 samples in the low-risk group. We then performed immune checkpoint analysis to investigate the potential precision application of ICIs in BRCA patients. As shown in Figure 5c, the analysis revealed significant differences between the high- and low-risk groups. Noteworthily, the high-risk group indicated lower expression levels of CD200, NRP1, and TNFRSF14, suggesting that tumor cells might evade immune surveillance through inhibiting the function of these immune checkpoint genes, which could subsequently affect patient prognosis.

Further examination of the tumor microenvironment (TME) indicated that the high-risk group had significantly lower stromal and ESTIMATE scores (the latter combining both stromal and immune components) compared to the low-risk group. This finding shows that the high-risk tumors possess a higher purity and a more active TME, a characteristic that may be associated with immune evasion and tumor progression (Figure 5d). Moreover, GSEA presented in Figure 5e demonstrated distinct enrichment patterns between the two risk groups. The low-risk group was enriched in metabolism-related pathways, such as fatty acid metabolism and glycogen synthesis, whereas the high-risk group was predominantly enriched in pathways related to tumor metastasis, immune response, and cell cycle regulation. These results show that the high-risk group may influence patient outcomes by modulating tumor immune evasion, metastasis, and cell cycle dynamics.

### 3.6. Differences in Chemotherapeutic Sensitivity Between High- and Low-Risk Groups

**Figure 6:**
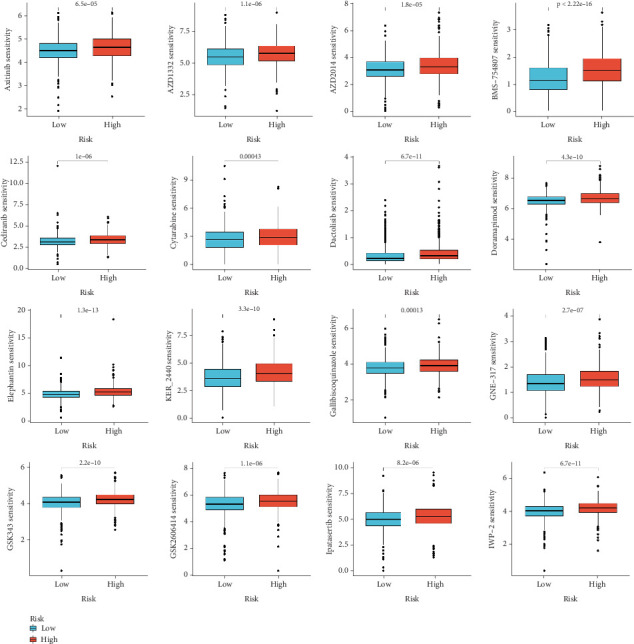
Drug sensitivity analysis. Boxplots showcasing the comparative drug sensitivity (IC50, half-maximal inhibitory concentration) across the high- and low-risk groups, revealing potential therapeutic responses.

The analysis of box plot data reveals significant differences in chemotherapeutic sensitivity between the high- and low-risk groups (Figure 6). For example, patients in the high-risk group exhibit markedly reduced sensitivity to agents such as axitinib, cisplatin, and doxorubicin compared to those in the low-risk group. This disparity may arise from intrinsic tumor characteristics—including gene expression patterns and variations in the TME—that contribute to greater drug resistance or more aggressive tumor phenotypes in high-risk patients. In contrast, low-risk patients generally show enhanced sensitivity to most of the drugs tested. The statistical significance of these differences (as indicated by *p* values) further validates this stratification and underscores the need for personalized therapeutic strategies.

### 3.7. Causal Impact of TBD52 Gene Variants on BRCA Risk

**Figure 7:**
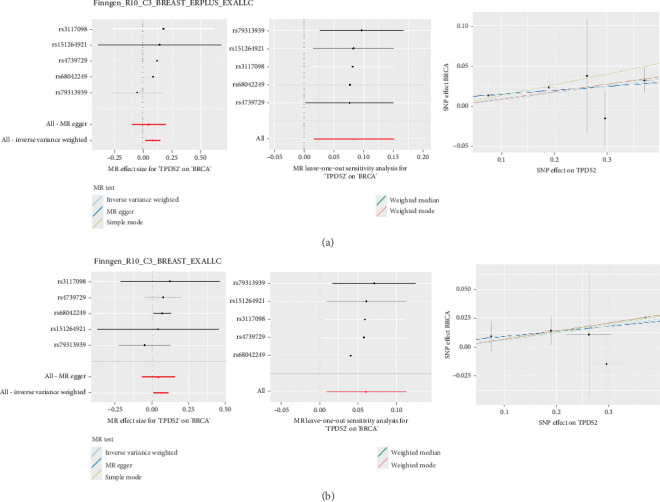
Integration of Mendelian randomization analysis demonstrating the oncogenic role of TPD52 in breast cancer. (a) The upper panel shows results from Finngen_R10_C3_BREAST_ERPLUS_EXALLC dataset. (b) The lower panel shows results from Finngen_R10_C3_BREAST_EXALLC dataset. Each panel consists of three plots: Left plots display MR effect sizes for ‘TPD52' on ‘BRCA' with different SNPs (rs3117098, rs15126492, rs4739729, rs68042249, and rs79313939) and aggregated estimates (All-MR egger and All- inverse variance weighted). Middle plots show MR leave-one-out sensitivity analysis for ‘TPD52' on ‘BRCA'. Right plots illustrate SNP effects on TPD52 versus BRCA with different statistical methods (inverse variance weighted, MR egger, simple mode, weighted median, and weighted mode) represented by different colored lines. Error bars represent confidence intervals. The *x*-axis in all right plots represents SNP effect on TPD52, while the *y*-axis shows SNP effect on BRCA.

Figure 7 presents the results of our MR analysis, which evaluates the relationship that determines causality between genetic variants in the TBD52 gene and the risk of BRCA. Using a panel of diverse single-nucleotide polymorphisms (SNPs) as instrumental variables, the analysis consistently indicates that increased expression or activity of the TBD52 is causally linked with an elevated risk of developing BRCA. Each of the evaluated SNPs contributed to estimating this relationship, and their combined effects support the notion that genetic modulation of TBD52 may play a significant part in the pathogenesis of BRCA.

These findings were robust across various MR methodologies, minimizing the potential impact of confounding factors and reinforcing the reliability of this causal inference. The evidence provided by this analysis emphasizes the importance of the TBD52 gene as a prospective marker for BRCA susceptibility and recommends that it may act as a promising target for future therapeutic interventions aimed at reducing BRCA risk.

### 3.8. Investigating the Role of TPD52 Through In Vitro Experiments

According to the above findings, there is a strong possibility that an increased TPD52 expression is connected to a negative prognosis in breast carcinoma. To further grasp the meaningfulness of TPD52 in breast carcinoma, the association between TPD52 expression and biological role was explored.

**Figure 8:**
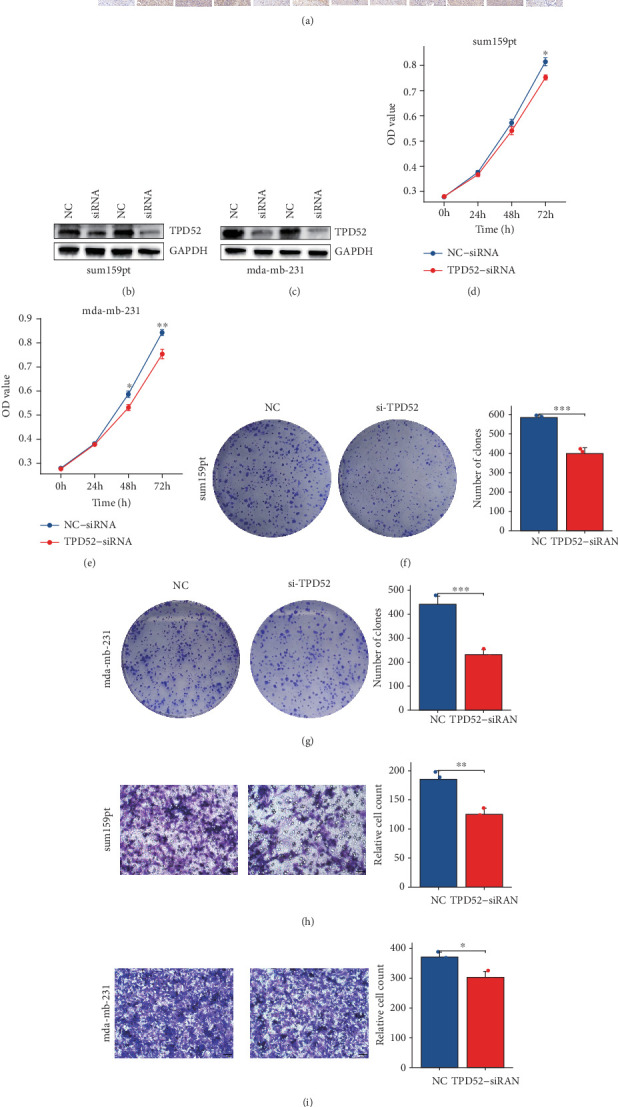
(a) IHC analysis shows the TPD52 protein expression levels in TNBC (Tumor) or the paired nontumor tissues (Normal), (scale bar : 50 *μ*m). (b, c) Knocking out the TPD52 in breast carcinoma cell lines by siRNA. Assessing the effects of TPD52 by (d, e) CCK8 and (f, g) colony formation. (h, i) Assessment of migration by Transwell assay.

The results of IHC exhibited the TPD52 was expressed at higher levels in TNBC compared with corresponding normal paired nontumor tissue (Figure 8). To further explore the function of TPD52 in SUM159PT and MDA-MB-231, TPD52 protein expression were reduced by siRNA (Figure 8). CCK8 (Figure 8) and colony formation assays (Figure 8) showed that silencing TPD52 reduces cell proliferation, while Transwell assays (Figure 8) indicated that it inhibits cell migration in SUM159PT and MDA-MB-231 cell lines.

## 4. Discussion

Global and national cancer statistics underscore that the burden of BRCA remains formidable. In 2022, an estimated 19.96 million new cancer cases were reported worldwide, of which approximately 2.31 million (11.6%) were BRCA cases. Among malignant tumors in women, BRCA ranks second only to lung cancer (12.4%). Globally, there were about 9.74 million cancer-related deaths in the same year, with BRCA accounting for roughly 660,000 deaths (6.9%), ranking it as the fourth most common cause of cancer-related deaths. In China, 2022 witnessed approximately 4.825 million new cancer cases, including around 357,000 BRCA cases (ranked sixth among all cancers), and about 2.574 million cancer deaths, with BRCA responsible for roughly 75,000 deaths (ranked seventh) [44]. These data indicate that BRCA—and particularly TNBC—poses substantial risks in terms of both incidence and mortality. TNBC is defined by the absence of PR, ER, and HER2. It is featured by rapid growth, high metastatic potential (notably to the lung, liver, and brain), and, despite often showing an initial favorable response to chemotherapy, a high rate of relapse and poor prognosis [45]. Its 5-year relative survival rate is around 77%, significantly lower than the over 90% observed for BRCA with hormone receptor–positive [46]. The lack of specific targeted therapies further limits clinical treatment options for TNBC, underscoring the ongoing need to develop more effective immunotherapeutic and molecular-targeted strategies.

The singular dual role of MECs in the BRCA microenvironment is highlighted in this study. Under normal physiological conditions, MECs act as structural barriers and secrete tumor-suppressive factors that help maintain LEC polarity and tissue integrity, thereby impeding tumor initiation and invasion [47, 48]. However, our results indicate that MECs in TNBC undergo significant functional reprogramming, characterized by dramatic gene expression and phenotypic changes. Specifically, genes associated with cellular stress, immune responses, and proliferation—including FDCSP, KRT14, and KRT17—were significantly upregulated in MECs from TNBC tissues, while tumor-suppressive genes related to the extracellular matrix (ECM) remodeling and inflammation control—such as CXCL8, SRGN, and DCN—were downregulated [44, 49–54]. These transitions underscore a critical shift in the biological role of MECs, from acting as tumor-suppressive barriers to becoming facilitators of tumor progression.

Such functional reprogramming likely contributes to the TNBC microenvironment's aggressive behaviors, given MECs' emerging role in regulating leukocyte infiltration, *αβ* T cell activation, and tumor invasion. The observed genomic instability of MECs—validated by our CNV analysis—further supports the hypothesis that MECs may evolve alongside tumor cells, adopting protumorigenic properties under selective pressure. Additionally, pseudotime trajectory analysis demonstrated distinct dynamic transitions in cellular phenotypes, suggesting that MECs may actively participate in tumor progression by modulating their functional states. In this regard, targeting MEC-mediated pathways may represent a robust therapeutic opportunity for controlling TNBC aggressiveness and metastasis.

Previous studies have elucidated the development of prognostic models based on BRCA-related pathways, demonstrating good predictive value [39]. Using multiple algorithmic evaluations, our CoxBoost model demonstrated optimal performance across several cohorts, yielding the highest mean AUC values for 1-, 3-, and 5-year survival predictions. Univariate Cox regression and subsequent meta-analysis confirmed that the model reliably stratifies patients into the low- and high-risk groups across 20 survival cohorts from 12 datasets (HR = 1.2730, 95%CI = 1.1324–1.4312, *p* < 0.001).

Our study also sheds light on the immunological mechanisms underpinning TNBC progression and therapy resistance. Immune microenvironment analysis revealed that high-risk patients exhibit downregulated expression of key genes of immune checkpoint such as CD44 and TNFRSF14, which may compromise antitumor immune responses [55, 56]. This may enable tumor cells to escape immune system monitoring and promote immune tolerance within the TME—contributing to the poor outcomes observed in the high-risk groups. Furthermore, high-risk patients demonstrated reduced stromal and immune scores and exhibited enrichment in prometastatic and proliferation-related pathways, such as those involved in DNA repair and cell cycle regulation [48]. These results highlight the pivotal role of immune evasion in TNBC progression and suggest that immune checkpoint blockade therapies may be less effective in high-risk patients due to intrinsic TME constraints. Pathway enrichment analyses further demonstrated that while the low-risk group is predominantly correlated with metabolic pathways (e.g., drug metabolism and MAPK signaling), the high-risk group shows notable enrichment in pathways involved in the DNA replication and cell cycle. These findings suggest a link between the malignant phenotype in high-risk patients and uncontrolled proliferation as well as genomic instability. Additionally, the high-risk group exhibited widespread resistance to 16 chemotherapy drugs, consisting of axitinib and dasatinib, thereby further elucidating the basis for their poor prognosis. Our findings emphasize the importance of comprehensive risk stratification to inform personalized therapeutic strategies, enabling high-risk TNBC patients to benefit from alternative treatment approaches, such as combination therapies targeting immune evasion pathways or metabolic vulnerabilities.

Through MR analysis, we identified TPD52 as a gene significantly associated with BRCA risk; its expression quantitative trait locus (eQTL) effect may promote tumorigenesis by modulating cell cycle progression. Building on this genetic evidence, in vitro trials were conducted to further explore the functional role of TPD52 in BRCA. IHC analyses revealed that TPD52 is notably overexpressed in TNBC tissues compared to nearby normal tissues, suggesting its involvement in TNBC progression. To elucidate its biological role, TPD52 expression was silenced via siRNA in TNBC cell lines (MDA-MB-231 and SUM159PT). TPD52 knockdown resulted in a notable reduction in cell proliferation—as shown by CCK8 and colony formation assays (*p* < 0.001)—and markedly decreased cell migration, as demonstrated by Transwell assays (*p* < 0.01). These data directly indicate that TPD52 drives TNBC progression by modulating both proliferative and invasive phenotypes, consistent with its association with high-risk profiles in our prognostic model.

Overall, these findings provide compelling evidence that both the altered state of MECs and the overexpression of TPD52 contribute to the aggressive nature of TNBC, offering potential targets for future therapeutic interventions.

## 5. Conclusions

Overall, this study established a prognostic risk scoring model that effectively stratifies triple-negative patients, assesses prognosis, and predicts immunotherapy responses. This model provides clinicians with a valuable tool for devising personalized treatment plans, particularly by identifying patients who are most likely to benefit from immunotherapy, thereby potentially improving survival outcomes. Furthermore, our findings reveal that the signature gene TPD52 is significantly upregulated in patients with shorter survival, suggesting that it could serve as a promising therapeutic target and pave the way for novel clinical interventions.

## Data Availability

The study's dataset is available in the online knowledge base, with the repository name and login number detailed in the article.
